# Patient satisfaction and scope size of flexible ureteroscopy for upper urinary stones: A comparative study, multivariate analysis and subjective and objective evaluations

**DOI:** 10.1002/bco2.70231

**Published:** 2026-05-18

**Authors:** Ihab A. Hekal, Yousef I. Hekal, Jana I. Hekal, Abdulla Al Memari

**Affiliations:** ^1^ Urology Department Ain Al Khaleej Hospital Al Ain United Arab Emirates; ^2^ Mansoura Faculty of Medicine Mansoura Egypt

**Keywords:** DJ, endoscopy, flexible ureteroscopy, LASER, patient satisfaction, scope size, ureteric stent

## Abstract

**Objective:**

This study aimed to evaluate subjectively and objectively the use of the slimmest scope, 7.5 Fr, on patient quality of life and outcome for upper urinary stones (UUS) treated by flexible ureteroscopy and to study the impact of ureteric stent insertion and operative techniques on patient satisfaction.

**Methods:**

Between January 2024 and October 2025, 120 cases with UUS met our inclusion criteria: stone burden > 7 mm (single or multiple), radio‐opaque stone, no UTI and not more than mild hydronephrosis preoperatively. Two endoscopic techniques were encountered. An equal number of cases were operated on using a scope of 7.5 Fr (Group A) and 9.5 Fr (Group B). The comparisons of patient factors included age and body mass index (BMI). Stone size, number and laterality were analysed. Operative factors included ureteral dilatation, stent insertion and procedure duration. Post‐operative stent, its duration, pain level, dark urine (possible significant haematuria) or dysuria (possible infection), retained stone or other post‐operative complications were assessed.

**Results:**

Both scope groups were demographically insignificant. The stone location and side were comparable. Operative details had a nearly insignificant comparison. Group A had a long operative time. Stent insertion was higher in Group B. Subjectively, the dark urine symptom was reported in 37 and 23 cases in Groups A and B, respectively (*p* < 0.05). Interestingly, 11 cases needed opioid injections for post‐operative colic in Group A (*p* = 0.008). The stone‐free rate was 89.2% and 91.7% in Groups A and B, respectively (*p* = 0.487). All stented cases had mild storage lower urinary symptoms. No major surgical complications were reported.

**Conclusion:**

Scope size has no impact on patient post‐operative outcome. Interestingly, a long operative time with a small scope was observed. Ureteral dilation and stent insertion cases have urinary post‐operative symptoms. A nonstent procedure is advised in noncomplicated cases (Hekal's criteria).

## 1 INTRODUCTION AND AIM OF THE WORK

Urolithiasis is defined as a disease diagnosed by the presence of one or more stones in the urinary tract, with an approximate recurrence rate of 30%–50% within 5 years.^1^ Treatment modalities have changed over decades to reach a reliable and tolerable procedure.^2^ Stone recurrence makes those patients vulnerable to repeat surgical or endoscopic procedures. Based on this fact, the need for a safe, liable and repetitive procedure is indicated.

Since the implementation of disposable flexible ureteroscopy (FURS) in urology practice, it has become highly used worldwide. Upper urinary stones are the most common practice for FURS (European Association of Urology [EAU] Guidelines 2025). The manufacturing companies have innovated a small diameter with better scope imaging and LASER technology to improve urology work. The aims of the slimmest scope are to reduce operative time, facilitate return to work and improve quality of life post‐operatively.^3^

Sufficient data and conclusions regarding patient perception, value and satisfaction with the operation with a slim scope are less encountered in the literature. Subsequently, the recommended operative practice and possible nonstented procedure regarding patient outcome are still debatable and nonconclusive.

In our study, we focused on the patients' impact from their own views, such as urine symptoms, post‐operative pain and lower urinary symptoms. Objective assessments were urine analysis, pain score and control X‐ray. Analyses of different factors, including scope size and surgical common practice techniques, were studied. Stone clearance and stent insertion with their related complications were considered.

## 2 PATIENTS AND METHODOLOGY

Among 436 ureteroscopy procedures for urinary stones that had been performed between January 2024 and October 2025 in our centre, 120 cases with upper urinary stones were included in our study.

Inclusion criteria were stone burden of more than 7 mm (single or multiple), radio‐opaque stone, location in the renal or upper ureter, no preoperative UTI and not more than mild hydronephrosis on preoperative imaging.

Exclusion criteria included congenital renal and ureteric anomalies, solitary kidney, children, lucent stone, septic condition, prestented cases, post‐extracorporeal shock wave lithotripsy (ESWL) cases, intraoperative ureteric injury and severe urinary obstructive uropathy.

All cases were done under general anaesthesia, and a preoperative antibiotic was given. Two highly skilled surgeons were encountered randomly. Surgeon 1 endoscopic technique included no ureteral dilatation unless indicated, using an access sheath of 10–12 Fr and scope water irrigation pressure of not more than 40 mmHg. Surgeon 2 endoscopic technique included routine ureteral sequential dilatation up to 12–14 Ch, using an access sheath of 12–14 Fr and scope water irrigation pressure of more than 40 mmHg. Post‐operative pain medication was given. The Lumenis Pulse™ 120H Holmium Laser System Technology was used for stone breakage and dusting. Both scopes were supplied by the same manufacturing company.

Cases were operated using FURS 7.5 and 9.5 Fr in both surgical groups without prior selection (nonrandomized). In all cases, stone dusting was the main mode used with LASER. Post‐operative control by kidney–ureter–bladder (KUB) X‐ray was performed. Post‐operatively (after 1 week), patients were subjectively asked about renal colic pain, burning urinary sensation, dark‐coloured or bloody urine, urgency and frequency. Objectively, cases were tested for pain score scale chart, urine analysis (haematuria and/or pyuria) and KUB X‐ray for residual stone or stent migration.

The comparisons of different factors were assessed including patient factors: age and body mass index (BMI). Stone site, number and laterality were analysed. Operative factors included ureteral dilatation and duration of procedure. Post‐operative factors included stent, its duration, subjective pain level (either controlled on ordinary pain medications or requiring opioids: visual pain score > 3), presence of dark urine (possible significant haematuria), severe dysuria (possible urinary infection), redo endoscopic procedure for retained stone or other post‐operative complications.

For the statistical analyses, SPSS 16.0 for Windows (SPSS Inc., Chicago, IL, USA) was used. The chi‐square test was used to evaluate categorical variables. The independent *t*‐test for continuous variables was used for comparisons between groups. Statistical significance is considered for a *p* < 0.05. Multivariate analysis for tested variable was conducted.

## 3 RESULTS

Between January 2024 and October 2025, 120 cases met the inclusion criteria. The patients were divided into two equal groups according to scope size (Group A: FURS 7.5 Fr and Group B: FURS 9.5 Fr).

Demographic data are shown in Table 1, showing insignificant and comparable features of age, gender and BMI. The stone location and laterality were insignificant in both groups. All cases were done as 1‐day surgery, and no extra‐hospital stay had been encountered in our cohort.

**TABLE 1:** Demographic data: Group A using FURS 7.5 Fr and Group B using FURS 9.5 Fr.

		Group A (total no. 60)	Group B (total no. 60)	*p* value
Age (mean ± SD)		38.6 ± 11	39.6 ± 11.6	0.602
Gender	Male (% of total)	32 (26.7)	38 (31.7)	0.267
	Female (% of total)	28 (23.3)	22 (18.3)	
BMI (mean ± SD)		27.9 ± 4.75	28.2 ± 5.5	0.703
Stone size (mean ± SD)		10.24 ± 4.7	8.8 ± 3.4	0.0602
Stone side	Right (group %)	32 (49.2)	33 (50.8)	0.855
	Left (group %)	28 (50.9)	27 (49.1)	
Stone site	Renal (group %)	42 (58.3)	30 (41.7)	0.025
	Ureter (group %)	18 (37.5)	30 (62.5)	
Stone number	Single (group %)	48 (46.2)	56 (53.8)	0.032
	Multiple (group %)	12 (75)	4 (25)	

Abbreviations: BMI, body mass index; FURS, flexible ureteroscopy.

Table 2 shows the operative difference in both groups. Surgical endoscopic techniques were equal procedures in both groups. Ureteral dilatation was performed in 24 and 25 cases of Groups A and B, respectively (*p* value = 0.853). The surgical time was less in Group B (mean 44.08 min, *p* value = 0.128). The ureteric stent insertion was indicated in 47 and 55 cases of Groups A and B, respectively (*p* value = 0.041). Mean ureteric stent duration showed an insignificant difference. All stented cases had mild storage lower urinary symptoms, and no medications were needed. No surgical complications were reported in the groups, including ureteric injuries, renal extravasation or significant intrarenal haematuria.

**TABLE 2:** Operative findings based on scope size.

		Group A (total no. 60)	Group B (total no. 60)	*p* value
Surgeon	1	31	29	0.715
	2	29	31	
Ureteral dilatation (% of total)		24 (20)	25 (20.8)	0.853
Surgery time (mean ± SD)		49.75 ± 22.8	44.08 ± 17.1	0.128
DJ insertion (% of total)		47 (46.1)	55 (53.9)	0.041
DJ period (mean ± SD)		7.8 ± 6.03	9.3 ± 5.2	0.147

Abbreviation: DJ, double‐J.

Table 2 shows the post‐operative factors (as per patient complaints on subsequent visit): dark urine, annoying dysuria and/or unbearable renal colic. Dark urine (urine analysis shows insignificant gross haematuria < 50 RBCs/HPF) was demonstrated in 37 and 23 cases in Groups A and B, respectively (*p* value < 0.05). Dysuria (on urine analysis, pus cells < 10 pus cells/HPF) shows an insignificant difference in comparison. Interestingly, 11 cases needed opioid injections for intractable post‐operative renal colic in Group A (*p* value < 0.05). In our total cases, there were no readmissions for significant haematuria, urinary sepsis and/or severe renal colic. All cases were classified as Grade 1 of the Clavien–Dindo classification. Retained fragments were documented in 10.8% and 8.3% of Groups A and B, respectively (*p* value = 0.487).

Interestingly, we compared both surgical techniques to all previous variables (Table 3). All stone‐related parameters were insignificant. However, ureteral dilatation and stent fixation were significantly higher in Surgeon 2. Operative time was significantly less in Surgeon 1 (mean 42.75 min). Post‐operative parameters of renal pain and dark urine were insignificantly similar in both endoscopic techniques. On the other hand, 13.3% of the Endoscopic 2 group had retained fragments compared to 5.8% in the Endoscopic 1 group (*p* value = 0.037).

**TABLE 3:** Post‐operative assessment based on scope size.

	Group A (total no. 60)	Group B (total no. 60)	*p* value
Microscopic haematuria/dark urine (% of total)	37 (28.3)	23 (19.2)	0.044
Pyuria/dysuria (% of total)	14 (11.7)	15 (12.5)	0.831
Pain–ER visit (% of total)	11 (9.2)	2 (1.7)	0.008
Second‐look URS (% of total)	13 (10.8)	10 (8.3)	0.487

Abbreviations: ER, emergency room; URS, ureteroscopy.

Furthermore, we tried to study the ureteric stent against different variables. Table 4 shows that 102 cases needed ureteric stent fixation versus 18 nonstented cases of the total study group. BMI was insignificantly less in the nonstented group (mean 26.9). Single stone was the majority in the groups (*p* value = 0.764). Mean stone size was smaller (mean 8.75 mm) in the nonstented group (*p* value = 0.392). A significantly shorter operative time was observed in the nonstented group (mean 34.4 min, *p* value = 0.004). Almost all nonstented cases were operated under Endoscopic Technique 1 (*p* value < 0.05). Although 13 out of 18 nonstented cases were operated on using the 7.5 Fr scope size, this had a fair significant impact on group comparison. Regarding all tested post‐operative parameters, the nonstented group was less significant. Only one case had residual stone in the nonstented group (*p* value = 0.112).

**TABLE 4:** Comparison according to surgical endoscopic procedures.

		Surgeon 1 (total no. 60)	Surgeon 2 (total no. 60)	*p* value
Stone number (% of total)	Single	51 (42.5)	53 (44.2)	0.591
	Multiple	9 (7.5)	7 (5.8)	
Stone site (% of total)	Renal	36 (30)	36 (30)	1
	Ureter	24 (20)	24 (20)	
Stone side (% of total)	Right	28 (23.3)	37 (30.8)	0.099
	Left	32 (26.7)	23 (19.2)	
DJ insertion (% of total)		43 (35.8)	59 (49.2)	<0.005
Ureteral dilatation (% of total)		4 (3.3)	45 (37.5)	<0.005
Stone size (mean ± SD)		9.14 ± 3.3	9.9 ± 4.8	0.310
Surgery time (mean ± SD)		42.75 ± 19.16	51.0 ± 20.7	0.024
DJ period (mean ± SD)		7.9 ± 5.9	9.2 ± 5.4	0.232
Post‐operative dysuria (% of total)		18 (15)	11 (9.2)	0.136
Post‐operative dark urine (% of total)		31 (25.8)	26 (21.7)	0.361
Post‐operative severe pain–ER visit (% of total)		8 (6.7)	5 (4.2)	0.378
Second‐look URS (% of total)		7 (5.8)	10 (13.3)	0.037

Abbreviations: DJ, double‐J; ER, emergency room; URS, ureteroscopy.

Finally, we performed a multivariate analysis on cases with residual fragments (23 cases) that needed a second intervention with different studied factors. In our cases, we had a stone‐free rate (SFR) of 89.2% in the small‐scope group, whereas the rate was 91.7% in the large‐scope group. Residual stone cases were identified in 13 cases of FURS 7.5 Fr and 10 cases of FURS 9.5 Fr. Preoperative factors comparison of both scope groups showed that BMI (lower in small scope, mean 26.27 ± 2.46) and stone location (renal location in small scope) were significant, with *p* values of 0.001 and 0.022, respectively. The size of stone was insignificant in comparison between 7.5 and 9.5 Fr (means 15.8 and 12.3 mm, respectively, *p* value = 0.139).

On operative tested factors for residual stone cases, surgical time was shorter insignificantly on larger scope (10 min less, mean 58.5 ± 18 min, *p* value = 0.410). Surgical techniques had an insignificant difference in comparison between scope sizes (*p* value = 0.382). Stent insertion was comparable on comparison (*p* value > 0.05).

Among residual stone cases, all tested post‐operative factors, namely, haematuria, pyuria, pain and stent duration, were comparable in both scope groups (*p* value > 0.05).

However, in the stone‐free cases (97 cases), the scope size was an insignificant factor in the comparison. The median operative time was 40 min (the mean was 3 min less in FURS 9.5 Fr, *p* value = 0.292). Stone size was comparable in both scope groups (median 8 mm, *p* value = 0.214). Among nonstented, stone‐free cases (17 cases), procedure time was significantly shorter regardless of the used scope (mean 33.2 min, *p* value = 0.006). Interestingly, the mean BMI was 27 in this group (*p* value = 0.453).

## 4 DISCUSSION

Nowadays, FURS is a cornerstone in minimally invasive urology. The emergence of single‐use ureteroscopy addresses challenges associated with reusable devices, such as cross‐contamination, maintenance costs and degradation over time.^3^ Later, the slimmest scope, 7.5 Fr, with its vision, deflection and manoeuvrability comparable to those of the 9.5 Fr scope,^3^, ^4^ aimed to improve patient outcome without hindering therapeutic value.

Most studies compare technical aspects, vision, operative time, stone clearance and LASER power consumption. A group of authors found that 7.5 Fr in the treatment of kidney stones has higher lithotripsy efficiency and a lower complication rate compared to 9.5 Fr.^5^ The Geavlete et al. group recommended 7.5 Fr; no ureteral damage and 1‐day surgery are the main real minimally invasive characteristics of this scope.^6^ Recently, the scope became thinner, at 6.3 Fr, and was technically comparable to 7.5 Fr.^7^

In our study, we look from the patient's side. Patient dissatisfaction could reduce the choice of FURS as a treatment modality for patients. Those patients have a tendency for stone recurrence due to the nature of the disease. Our aim in this work is to study the impact of the slimmest (7.5 Fr) scope on patient quality of life, outcome and patient satisfaction. We focus on patient satisfaction post‐operatively from the subjective side (the patients' own words). Furthermore, objective factors could alter patient concerns about FURS, including SFR, operative time, residual stone and stent insertion (with the subsequent need to redo cystoscopy or ureteroscopy for its removal).

In our study, we selected a subjective assessment of distressing urological symptoms (as per patient complaint): dark urine, burning urine sensation, urgency, frequency and intractable renal pain. In our study, we preferred the assessment of uro‐related complaints rather than the psychometric assessment by Freiburg's questionnaire, which was studied in the literature before.^8^, ^9^

Objectively, we checked our patients with urine analysis to check for the presence of haematuria and pyuria as causes of dark urine and dysuria in their post‐operative complaints, with pain score assessments, as well as the need for opioids as an indication of severe colic pain. A post‐operative KUB X‐ray for the assessment of stone clearance had been done.

Our study is nonrandomized regarding scope size. We aimed to alleviate study bias and to reach reliable results for common urology practice; comparable, equally tested models have been chosen. The most common endoscopic practices were performed on equal cases randomly. The stone size was nearly comparable to that in common urology work. The same applies to the LASER machine and the mode of stone disintegration. Scope supply was from the same manufacturing company. Selection of radio‐opaque stones, which are mostly nearly similar in chemical composition: calcium oxalate (most of our cases had stone chemical analysis), was performed. All cases were 1‐day surgeries, and no major complications required an extra‐hospital stay.

Our results show that the slimmest scope (7.5 Fr) was not superior regarding post‐operative patient complaints. Furthermore, microscopic haematuria/dark urine and pain episodes were significant with the smallest scope. This could be explained by the longest operative time in this group. Even more, there is an insignificant SFR between both scope sizes, whereas in the literature, a higher rate is observed with the smallest scope (6.5 Fr), with a cumulative SFR of 91% in a systematic review (European Guidelines 2025).

With different common endoscopic techniques discussed in our study, both are comparable on the tested patient‐related factors with no significant differences. Although there was a relatively shorter operative time, less stent duration and a higher stone clearance rate in Surgical Endoscopy Technique 1, these differences were statistically insignificant.

Since its introduction in 1974, the ureteral access sheath (UAS) has been widely used with FURS, primarily because it facilitates the drainage of intra‐ureteral fluids, preventing injuries from high renal pelvic pressure.^10^, ^11^ Interestingly, there is no relation between SFR and the use of the access sheath.^12^, ^13^ Furthermore, ureteral dilatation and the access sheath also increase surgical costs and may injure the ureteral wall.^14^ FURS without an access sheath seems to be as safe as FURS with a sheath but requires less ancillary procedures. An additional advantage of skipping the ureteral dilation step is a shorter operative time.^15^ We emphasized that ureteral instrumentation—dilatation, UAS, prolonged procedure and stent—could affect the ureteral mucosa microscopically. Even more, it could alter the blood circulation in the ureter due to prolonged internal mucosal compression and may be associated with minute injuries. Later, it may reflect on post‐operative outcome, likely haematuria, ureteral spasm and colic.

In our study, regardless of scope size (UAS technique with ureteral dilation or without), nondilatation reflected significantly higher stone clearance and less procedure time. However, regarding post‐operative complaints, it was almost insignificant. The robust finding is that ureteral dilation does not affect patient post‐operative outcome (Table 4). In our cohort, 75% of the ureteral dilatations required stent insertion. In this group, with ureteral stenting due to dilation, patient satisfaction was affected in all post‐operative tested parameters (Table 5).

**TABLE 5:** Comparison according to ureteric stent insertion.

		With stent (total no. 102)	Without stent (total no. 18)	*p* value
Age		39.04 ± 11.8	39.7 ± 8	0.815
BMI		28.2 ± 5.3	26.9 ± 3.7	0.324
Stone number (% of total)	Single	88 (73.3)	16 (13.3)	0.764
	Multiple	14 (11.7)	2 (1.7)	
Stone site (% of total)	Renal	62 (4651.7)	10 (8.3)	0.676
	Ureter	40 (33.3)	8 (6.7)	
Stone side (% of total)	Right	60 (50)	5 (4.2)	0.015
	Left	42 (35)	13 (10.8)	
Ureteral dilatation (% of total)		48 (40)	1 (8)	0.001
Stone size (mean ± SD)		9.6 ± 4.2	8.75 ± 3.7	0.392
Surgery time (mean ± SD)		49.12 ± 20.7	34.4 ± 12.35	0.004
DJ period (mean ± SD)		10.03 ± 4.8	—	<0.05
Post‐operative dysuria (% of total)		21 (17.5)	8 (6.7)	0.029
Post‐operative dark urine (% of total)		44 (36.7)	13 (10.8)	0.023
Post‐operative severe pain–ER visit (% of total)		8 (6.7)	5 (4.2)	0.012
Second‐look URS (% of total)		22 (18.3)	1 (0.8)	0.112
Surgeon (% of total)	1	43 (35.8)	17 (14.2)	<0.005
	2	59 (49.2)	1 (0.8)	
Scope (% of total)	URS 7.5	47 (39.2)	13 (10.8)	0.041
	URS 9.5	55 (45.8)	5 (4.2)	

Abbreviations: BMI, body mass index; DJ, double‐J; ER, emergency room; URS, ureteroscopy.

Stone clearance is a fundamental issue for patient satisfaction. The SFR is different in the literature. Stone location, size, number or technique (e.g., T‐tilt position for lower calyceal stone) are variant factors affecting stone clearance.^16^ In a recent meta‐analysis, SFR ranged between 70% and 93%.^16^ In our study, it ranges between 89.2% in FURS 7.5 Fr and 91.7% in FURS 9.5 Fr in a single session. Even though the difference was insignificant in our study, it could rely on better image quality, deflection and manoeuvrability, augmented by the good irrigation flow of a larger scope.

An interesting finding was from the analysis of the stone‐free group; we found that scope size had no impact, and even more insignificantly, the large scope procedure time was shorter. The median size is 8 mm, and a median BMI of 27 is observed in the nonstented stone‐free group. Low BMI is linked to short procedure time (with high BMI, an expected difficult anaesthesia, patient positioning or technical factors, e.g., poor intraoperative imaging with thick adipose tissue or anatomical variation^17^).

Another interesting point is the use of the ureteral stent. Since 2011, a systematic review has revealed obvious disadvantages of ureteral stents after ureteroscopic lithotripsy, including lower urinary tract symptoms and pain. Stents do not improve SFR, fever, incidence of urinary tract infection, unplanned medical visits, requirement for analgesia or late post‐operative complications. Ureteral stenting after uncomplicated ureteroscopic lithotripsy could be unnecessary.^18^, ^19^ Even so, the majority of cases are still stented, with an impact on patient quality of life and the need for redo cystoscopy for stent removal.

Regarding ureteral stenting, some endoscopes rely on stone size, which is denied by Picozzi et al.: Stone diameter is not a variable in the preoperative or intraoperative decision process of placing or not placing a ureteral stent in patients undergoing uncomplicated FURS.^20^ A meta‐analysis study on ureteral stents showed an inability to conduct any of the preplanned subgroup analyses, in particular those based on stone size, stone location and use of ureteral dilation.^21^

There are many recommended criteria for nonstenting post‐FURS.^22^ A noncomplicated procedure and normal kidney and ureter conditions are considered an indication of a nonstent procedure.^23^ Some suggest stone size or operative time as factors for ureteral stenting decisions.^14^

In our study, the nonstented group had significantly less haematuria/dark urine, burning sensation or even pain episodes. Rather than that, the omission privilege of re‐treatment for stent removal or a second ureteroscopy of retained fragments is achieved.

To the best of our knowledge, there is no other similar article comparing the two sizes of scope regarding patient satisfaction parameters.

In brief, our study shows that a small scope has a longer operative time. With a small scope, the post‐operative stent is still indicated, regardless of the technique used. Moreover, a small scope is associated with more bothersome patient symptoms, such as dark urine and even pain episodes (that require opioids), which are more frequent. Large scopes are less likely to have retained fragments and have higher stone clearance than small scopes.

The American Urological Association (AUA) guidelines published criteria for uncomplicated ureteroscopy in 2025. To enhance patient satisfaction, we clinically correlated an exploratory hypothesis derived from the current data updated for nonstented FURS. In our study, based on our conclusion in scope size and used techniques, we recommended what we named ‘Hekal Criteria’: 5 Not's—stone of not more than 8 mm, BMI below or equal to 27, procedure time of not more than 40 min, UAS of not more than 9/10 Fr and not using ureteral dilators. Those criteria were derived from our conclusion of the stone‐free nonstented group analysis. We recommend adding 5 Not's criteria to be enrolled in guidelines after validation to enhance the selection of nonstented FURS for better patient satisfaction and outcome, provided complete stone disintegration.

To consolidate our conclusion and validate the suggested nonstent criteria, a multicentre, randomized prospective study on scope size is required. Further, a validated urinary questionnaire is required to evaluate post‐operative urology cases.

Finally, in our opinion, the scope size is not the only factor for improving patient satisfaction. The need for a small access sheath without compromising irrigation flow, impairing vision or affecting intrarenal pelvic pressure, rather than ureteral dilatation omission, will be of great value for the FURS procedure. Furthermore, this will positively impact the need for a stent, SFR and further improvement of patient quality of life and procedure satisfaction. We recommend a prospective study on our recommended nonstented criteria (5 Not's) to be validated in a high number of patients.

## 5 CONCLUSIONS

Among our selection criteria, on subjective and objective analysis, scope size has no benefit to the patient or their post‐operative outcome and satisfaction. Interestingly, a long operative time with a small scope was observed. The SFRs are nearly equal regardless of scope size.

Omission of the ureteral dilation step and stent insertion in FURS procedures will result in a calm and less symptomatic post‐operative patient outcome.

Different FURS endoscopic techniques have an indirect impact on patient post‐operative outcome (ureteral dilation and subsequent stent insertion).

Nonstent FURS is advised in noncomplicated cases, provided complete stone clearance. A suggested criterion for the nonstent procedure to enhance patient satisfaction (Hekal's 5 Not's criteria) is advised for validation.

## AUTHOR CONTRIBUTIONS

Ihab A. Hekal contributed as Surgeon 1 and participated in the study design, manuscript writing and statistical analysis. Yousef I. Hekal and Jana I. Hekal contributed to the research and data collection for Section 4. Abdulla Al Memari contributed through supervision of the study.

## CONFLICT OF INTEREST STATEMENT

The authors declare no conflicts of interest.

## Data Availability

Research data are not shared.
